# A single strand: A simplified approach to DNA origami

**DOI:** 10.3389/fchem.2023.1126177

**Published:** 2023-02-20

**Authors:** Micah Yang, David Bakker, Dyuti Raghu, Isaac T. S. Li

**Affiliations:** Department of Chemistry, The University of British Columbia, Kelowna, BC, Canada

**Keywords:** DNA origami, DNA nanostructure design, ssDNA, DNA nanotechnology, self-assembly, nanomachines

## Abstract

Just as a single polypeptide strand can self-fold into a complex 3D structure, a single strand of DNA can self-fold into DNA origami. Most DNA origami structures (i.e., the scaffold-staple and DNA tiling systems) utilize hundreds of short single-stranded DNA. As such, these structures come with challenges inherent to intermolecular construction. Many assembly challenges involving intermolecular interactions can be resolved if the origami structure is constructed from one DNA strand, where folding is not concentration dependent, the folded structure is more resistant to nuclease degradation, and the synthesis can be achieved at an industrial scale at a thousandth of the cost. This review discusses the design principles and considerations employed in single-stranded DNA origami and its potential benefits and drawbacks.

## 1 Introduction

While DNA originates as the cellular mechanism for long-term genetic information storage, its highly programmable nature and predictable secondary structure folding make it an excellent material for creating nanostructures. Using DNA as a building material was first suggested in 1982 (Seeman, 1982), and in the decades since, an entire field has emerged. Duplexed double-stranded DNA (dsDNA) forms a rod, that is, remarkably rigid at length scales shorter than its persistence length (L_p_ ∼ 50 nm) (Mitchell et al., 2017), while single-stranded DNA (ssDNA) is very flexible (L_p_ ∼ 2 nm) (Chi et al., 2013; Roth et al., 2018). This enables rigid dsDNA and flexible ssDNA segments to be woven together to form pre-designed 2D sheets and 3D structures known as DNA origami (Rothemund, 2006; Douglas et al., 2009). Additionally, DNA origami is not limited to a singular static state. Various control mechanisms can be employed to dynamically switch between different engineered conformations—for instance, opening and closing a box (Andersen et al., 2009). While DNA has become a versatile building material for nanostructures and nanomachines, construction architectures using hundreds of short ssDNA strands suffer from several drawbacks that limit its scalability.

**FIGURE 1:**
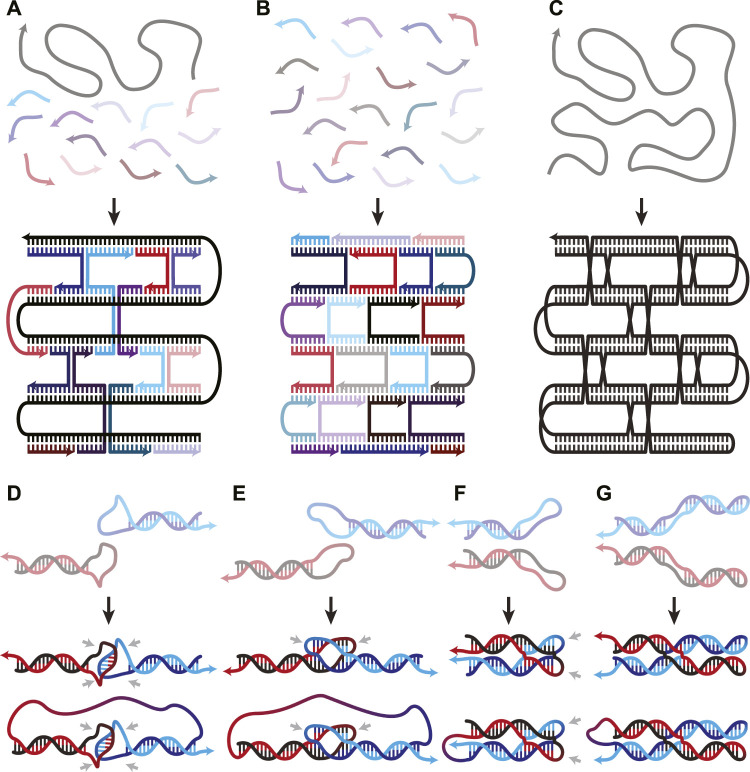
**(A–C)** Overview figure of DNA Origami design architectures, adapted from (Han et al., 2017). **(A)** Scaffold-staple: a long, singular scaffold strand runs throughout the entire origami and is folded into the desired structure by many short oligonucleotide staple strands. **(B)** Staple-only: the nanostructure is exclusively composed of short oligonucleotides. **(C)** Single-stranded: a singular strand of nucleotides folds and interacts with itself to form the nanostructure. **(D–G)** WC interactions in both inter- and intra-molecular DNA secondary structures. **(D)** Unstacked hairpin-kissing, where two loops bind without stem-stacking interactions. **(E)** Linear and **(F)** parallel bubble-bubble interactions, where two hairpins kiss while retaining stacking interactions with the stem. **(G)** Paranemic cohesion, where two duplexes contain unpaired loops along their lengths that hybridize. One duplex lies entirely on top of the other. Two final structures are shown in panels D-G; the upper and lower structures illustrate the same interaction occurring either inter- or intra-molecularly, respectively. Isolated grey arrows in D-G indicate regions which must remain unpaired in the final structures. Arrows in all figures indicate 5′→3′ direction.

Since Rothemund’s 2006 paper introducing the concept, DNA origami has predominantly been built using their scaffold and staples architecture (Rothemund, 2006). A long ssDNA runs throughout the structure, acting as the scaffold, while hundreds of short ssDNA hybridize and staple the scaffold into its designed shape (Figure 1A). Less extensively utilized schemes composed exclusively of short oligonucleotides (Figure 1B) have also been used to create nanostructures (Ke et al., 2012; Ong et al., 2017) such as hollow rods of various circumferences (Yin et al., 2008) and the alphabet (Wei et al., 2012). These multi-stranded DNA origami (msOrigami) architectures make excellent use of DNA’s innate programmability. Over the years, the field has seen significant development, from design software (Benson et al., 2015; Veneziano et al., 2016), to curved 3D and wireframe nanostructures (Ke et al., 2009; Han et al., 2011; 2013; Zhang et al., 2015; Wang et al., 2019a). Nevertheless, msOrigami’s main drawbacks result from its multi-stranded nature: it is difficult to effectively combine reagent strands to generate high product yield, the resulting structure has multiple free ends which make it vulnerable to enzymatic degradation in biological environments, and scaling its production to industrial levels remains cost-prohibitive. These problems can be solved by simplifying the design to a single ingredient, where intramolecular interactions within a single ssDNA fold itself into a complete nanostructure (Figure 1C), like a protein.

This review discusses available intramolecular DNA secondary structures and how they are used to construct ssDNA origami (ssOrigami). SsOrigami’s benefits are highlighted: the removal of concentration dependent folding, the improved *in vivo* stability of ssOrigami compared to msOrigami systems, and the reduced manufacturing cost at an industrial scale. An overview of existing proof-of-concept experiments that have taken advantage of these benefits to demonstrate ssOrigami’s potential is provided.

## 2 The secondary structural building blocks in ssOrigami

**FIGURE 2:**
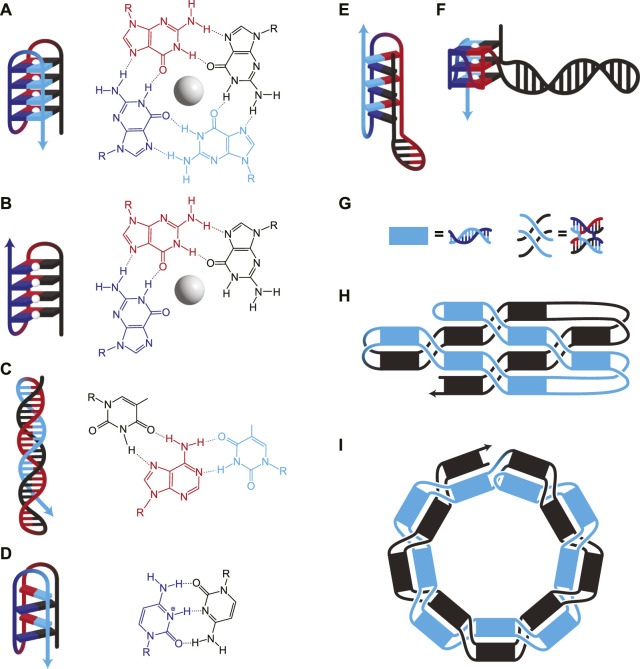
**(A–F)** DNA structures involving nWC interactions. **(A)** G-quadruplex, where four guanines interact to form a plane around a stabilizing monovalent (typically K^+^) cation (white sphere). Consecutive guanines enable multiple planes which stack for additional stability. **(B)** G-triplex, similar to G-quadruplex, but where only three guanines interact in each plane. **(C)** A Hoogsteen triplex, where a WC duplex is invaded by a triplex-forming strand. The triplex-forming strand (blue) is made entirely of pyrimidines and binds only to the “central” strand (red), which is made entirely of purines. The molecular structure shows interactions on a single plane, in this case, TA*T. **(D)** I-motif, where intercalating pairs of C^+^ and C bind. Hybrids of WC and nWC structures involving **(E)** an i-motif with a duplex bulge and **(F)** a G-quadruplex with a duplex bulge where a G-quadruplex has WC base pairing within one of its loops. **(G–I)** Unknotted *versus* knotted ssDNA nanostructures. **(G)** Duplexes and paranemic cohesions have been visually simplified for clarity in H and I. **(H)** A symbolic representation of the area-filling unknotted structure. The blue section lies entirely on top of the black section, which allows the folding of this structure to occur in two thermodynamically separate steps: First, the initial formation of the helices, then the formation of the paranemic cohesions. **(I)** A symbolic representation of the knotted structure. At each neighbouring paranemic cohesion, the duplexes swap sides. In H and I, the figure shows a small example, but the methods have been scaled to larger structures.

DNA secondary structures come in two flavours—Watson-Crick (WC) base pairing (Figures 1D–G), and non-WC base interactions (nWC) (Figures 2A–F). Several excellent reviews have focused on the characterization of each structure, including paranemic cohesion (Wang et al., 2019b), the G-quadruplex (Mukundan and Phan, 2013; Cheng et al., 2021), the i-motif (Abou Assi et al., 2018), and how they work in concert to form functional DNA origami and nanodevices (Rothemund, 2006; Saccà and Niemeyer, 2012; Tian et al., 2022). While these structures were typically demonstrated in systems with intermolecular interactions, they can form just as easily, if not more, intramolecularly in ssOrigami due to an effective increased local concentration and improved stoichiometric control.

### 2.1 Secondary structures based on Watson-Crick interactions

At the heart of all DNA nanostructures is the ability to bring two distinct sections of a DNA sequence together spatially. In msOrigami, this occurs intermolecularly between a staple and the scaffold or between multiple staples; in ssOrigami, it is entirely intramolecular. This usually occurs by having an unbound DNA sequence within a duplex, that is, complementary to another unbound sequence elsewhere in the structure. When these two regions touch, they hybridize, producing more complex secondary structures.

When two hairpin loops are complementary to each other, they form hairpin-kissing interactions. It is common to imagine hairpin-kissing as in Figure 1D, where base pairing occurs somewhere in the central portion of the loops. Such hairpin interactions with five complementary bases have been shown to have melting temperatures ∼29°C, which makes them difficult to use as structural components in DNA origami (Carr and Marky, 2019). Having longer stretches of complementary bases in each loop may seem like it would increase stability; however, there is an upper limit to the number of base pairs that can form between even fully complementary loops. This is due to topological constraints: any right-handed turns generated by the double-helix produce left-handed turns elsewhere on the loop (Romano et al., 2012). The interplay between this torsional strain and the thermodynamic stability of increased base pairing creates a free energy minimum at 14 base pairs for a pair of 20-nucleotide (nt) loops in simulations (Romano et al., 2012). In these simulations, the 14 pairs are not sequential—they instead form two sets of seven pairs flanking an unpaired region in the centre of each loop (Figures 1E, F); this is called a bubble-bubble interaction. This minimizes topological strain while maximizing stacking and base pairing interactions. Whether the two stems are linear (Figure 1E) or parallel (Figure 1F) depends on the loop sequence.

There is some experimental evidence to support these simulations, as two fully complementary 20-nt hairpin loops showed qualitatively fewer hairpin-kissing interactions than when the central 8-nt of each loop were non-complementary (Barth et al., 2016). This indicates that, to some extent, the availability of the central nucleotides competed with the more favourable structure, lowering binding affinity. When the central 8-nt remained complementary, but either side of the loops was mismatched, no stable hairpin-kissing interactions were observed. This supports the theory that separate binding regions are necessary to relieve torsional strain. Linear bubble-bubble interactions have been utilized in ssOrigami to generate kilobase scale structures (Qian et al., 2012; Zheng et al., 2021).

Pushing this idea in a different direction, one can also use paranemic cohesion (Figure 1G) to adhere multiple regions of DNA (Shen et al., 2004; Qi et al., 2018). Paranemic cohesion is similar to the bubble-bubble interaction, except the complementary unpaired regions occur in the middle of each duplex instead of at the ends. A series of these interactions binding two duplexes together along their lengths can thus be designed. At each crossing, one duplex lies entirely on top of one another, allowing them to be separated without opening either duplex. Paranemic cohesion can occur anywhere within a duplex, making them structurally versatile.

### 2.2 Secondary structures based on Non-Watson-Crick interactions

Secondary structures based on nWC interactions, such as G-quadruplexes, G-triplexes, i-motifs, and triple helices, can also be used to bring disparate strands together. While G-quadruplexes consist of G-rich sequences with four regions of adjacent G residues (Figure 2A), recently, G-triplexes with three regions of adjacent G residues have also been shown to form (Figure 2B) (Limongelli et al., 2013). Certain nWC-interaction based secondary structures, such as intramolecular triple helices (Figure 2C) and i-motifs (Figure 2D), form under acidic conditions. nWC structures are normally formed from a single contiguous sequence, but it is possible to design them with long loops between nWC interactions which themselves may contain their own secondary structures. Researchers have found that having a hairpin within the bulge of a G-quadruplex (Figure 2F) increases the melting temperature of the structure by ∼13°C (Ngoc Nguyen et al., 2020). A similar structure with an i-motif/duplex hybrid (Figure 2E) has also been shown to exist stably under physiological conditions (Serrano-Chacón et al., 2021).

While nWC interactions are generally less stable than WC-based secondary structures at physiological conditions, they are stabilized by various environmental factors. nWC interactions are rarely showcased as structural components of DNA nanostructures due to their dynamic nature—but this makes them ideal for designing responsive nanostructures which change conformation based on external signals. For instance, G-quadruplexes and triplexes require cations to form (Jiang et al., 2015; You et al., 2017; Chu et al., 2019; Nishio et al., 2021). This ionic dependency enables dynamic switching between conformations. Another study shows that Cu^2+^ allows the switching of an i-motif to a DNA hairpin (Day et al., 2015). pH can also be used to control secondary structures, since certain nWC interactions are pH dependent. I-motifs and triple helices involve protonated cytosine residues, so they unfold as cytosine deprotonates at high pH. This behaviour can be exploited to design DNA-based sensors that change conformation in response to pH (Surana et al., 2011; Patino et al., 2019; Park et al., 2022). Proven to function in <100-nt DNA sensors, this conformational control has also been utilized in dynamic DNA origami assembly (Yang et al., 2019). SsOrigami systems have the added advantage of forming nWC interactions intramolecularly, so even in the unfolded state, disparate elements are kept in proximity for rapid refolding in response to environmental changes. Controlling nWC secondary structures using external cues allows for the design of responsive ssOrigami, especially when paired with more static elements.

## 3 Designing ssOrigami

While dynamic secondary structures enable nanomachines, static secondary structures form the foundation of DNA origami’s structural components. After the development of the scaffold-staple system in 2006 (Rothemund, 2006), ssOrigami saw limited development for over a decade as research effort was poured into msOrigami. A 286-nt ssDNA tetrahedron from 2009 is one of the few constructions of this era (Li et al., 2009) alongside a 198-nt prism of ssDNA that probed the lower limits of 3D DNA origami size (He et al., 2013). As the field of msOrigami grew rapidly (Dey et al., 2021), ssOrigami at the kilobase scale remained elusive. These larger ssDNA nanostructures were challenging to synthesize due to the kinetic constraints of knotting: a singular strand threading back through a loop of itself is entropically disfavoured. Sequence design guidelines were developed in 2016 to minimize these energetic barriers and enable the design of spontaneous knot formation (Kočar et al., 2016). The most important of these is the ‘free-end’ rule which states that free ends are required at each consecutive folding step to form the correct structure. Should folding occur in the wrong order and the free end hybridizes too early, the free end is lost, and it becomes impossible to thread through the pre-folded structure. These rules were co-developed with a knotted 286-nt pyramid-shaped wireframe ssDNA nanostructure that showed proof-of-concept (Kočar et al., 2016).

A major breakthrough came in 2017 when a multi-kilobase ssOrigami was developed by avoiding topological entanglements entirely (Han et al., 2017). This new approach used paranemic cohesion coupled with single helical turns between each crossing (Figure 2G) to create structures as large as 10 kilobases (Han et al., 2017). The ssDNA sequence was designed to first form a partially paired filament driven by base pairing in the duplexed portions. Subsequently, paranemic cohesions drive the unfolded structure to fold in half at a central hinge as these bases hybridize, completing the nanostructure (Figure 2H). One half of the completed nanostructure lies on top (blue in Figure 2H) of the other half (black in Figure 2H). Not only were a wide variety of raster-filled shapes and sizes demonstrated to fold in high yield (74%–97% depending on the structure), but they were also capable of functionalization with various handles like biotin.

Combining the rules for ssDNA knotting with the paranemic cohesion of the knot-free assembly enabled the construction of knotted multi-kilobase ssOrigami (Qi et al., 2018). This design used a repeating motif of a helical domain followed by a paranemic cohesion. The ssDNA sequence first folded into a dsDNA filament with alternating paired (helical domain) and unpaired (paranemic cohesion) segments (Figure 2G). This partially paired filament then formed the designed paranemic cohesions between the unpaired regions, resulting in the fully paired, knotted ssOrigami. Following the free end rule, the paranemic cohesions were carefully engineered to fold sequentially based on melting temperature. The wrapping of strands around each other results in a knotted structure because the filament must thread back through itself in order for all of the paranemic cohesions to form (Figure 2I). Therefore, this knotting requires a wire-frame structure for free ends to pass through.

A variety of planar and 3D knotted structures with 9–57 paranemic cohesions (each driving a separate threading step) were designed and folded successfully (Qi et al., 2018). Nevertheless, this topologically threaded design is not without limitations. The major drawback is that each threading step in the structure has a misfolding rate of 4%–10% because of the entropic constraints of self-threading. This scales exponentially with the number of threading steps, so more topologically complex designs were found to have lower yield (Qi et al., 2018). A summary of existing ssOrigami nanostructures is provided in Table 1. Without additional breakthroughs enabling higher step-yields of threading, future ssOrigami designs will likely utilize predominantly unknotted structures but may introduce some threaded knots to access a more diverse range of 3D structures.

**TABLE 1:** Overview of existing ssOrigami structures and associated major developments in the field.

ssOrigami structure	Major development	Size (nt)	Year
Tetrahedron Li et al. (2009)		286	2009
Prism He et al. (2013)		198	2013
Various arbitrary 2D planes (e.g.,: rhombus, heart, smiley face, *etc.*) Han et al. (2017)	Multi-kilobase ssOrigami nanostructures	966–10682	2017
Various arbitrary 2D (e.g., square lattice) and 3D (e.g., pentagonal pyramid) wireframes Qi et al. (2018)	Kilobase scale knotted and 3D ssOrigami	1673–7500	2018
Triangle	DNAzyme integration	520	2021
Quadrangle Jia et al. (2021)		683	
Various 2D planar shapes Zheng et al. (2021)		264–1809	2021

While the benefits of using ssDNA in designing static nanostructures are being addressed, the realm of dynamic ssOrigami designed using these principles is largely unexplored. The benefits of using responsive secondary structures in msOrigami design have been partially exploited (Daljit Singh et al., 2018), though many dynamic DNA nanostructures rely instead on the introduction of external oligos to induce conformational response (Andersen et al., 2009; Wickham et al., 2012; Kuzyk et al., 2014; Marras et al., 2015). Thus far, environmentally responsive nanostructures have been limited to msOrigami, but the same ideas could apply to ssOrigami. Structural and dynamic secondary structures could be combined into a single, unified sequence, preserving the advantages of ssOrigami.

## 4 Discussion

### 4.1 *In Vivo* stability and folding concentration

SsOrigami is not just a novel architecture, but also has inherent benefits due to its unimolecular nature. One of these benefits is increased structural lifetime in the presence of nucleases. MsOrigami has been shown to begin degrading in fetal bovine serum after ∼2 h due to nuclease activity, limiting its *in vivo* applications (Hahn et al., 2014; Lacroix et al., 2019). While DNA origami, in general, curbs nuclease activity relative to linear dsDNA by reducing accessibility to interior strands (Mei et al., 2011), every free 5′ and 3′ end remains a potential attack site for exonucleases. This has been mitigated by chemically modifying the free ends of the origami (Conway et al., 2013; Lacroix et al., 2019) or coating the origami in a polymer or protein (Perrault and Shih, 2014; Ponnuswamy et al., 2017). However, these methods all involve extra modifications to the DNA origami structure; an alternative approach to reduce the number of vulnerable ends is to utilize fewer strands. By using a single strand, exonuclease activity is inherently reduced. SsOrigami degradation was compared to msOrigami degradation with similar surface-area/mass ratios, and it was shown that after an hour in 9 U/mL DNase I, ssOrigami only showed 10% degradation (Jia et al., 2021). In contrast, the msOrigami showed anywhere from 34%–100% degradation (Jia et al., 2021).

In addition to increased resistance to nuclease degradation, ssOrigami assembly is also concentration independent since the folding is solely intramolecular. MsOrigami requires an excess of staple strands to fold properly, as each strand must diffuse in solution to find its respective binding site (Dey et al., 2021). As such, msOrigami structures are generally folded at concentrations between 20 and 100 nM of the scaffold and 10–20 times more staples (Wagenbauer et al., 2017; Thomas et al., 2020; Dey et al., 2021; Shetty et al., 2021). SsOrigami, by contrast, has all interacting segments on just one strand, so there is no lower concentration limit for assembly—only a practical lower limit of the desired final amount. Taking advantage of this, ssOrigami experiments have used folding concentrations of 1–10 nM (Qi et al., 2018; Jia et al., 2021; Zheng et al., 2021). In principle, the unimolecular nature of ssOrigami could also be harnessed to achieve faster folding, improved yield, and simplified purification, although these potential benefits have yet to be directly tested (Han et al., 2017).

### 4.2 Cost of production and scalability

Staple oligonucleotides are economically produced with chemical synthesis, while kilobase length ssDNA used for scaffolds—or in the case of ssOrigami, the entire nanostructure—are not produced chemically for commercial sale. Because synthesis error rate increases as the synthetic ssDNA is lengthened, the amount of DNA with an incorrect sequence rapidly increases (Hughes and Ellington, 2017). These long ssDNAs are thus traditionally isolated from dsDNA, that is, amplified with PCR or *in vivo* (Bush et al., 2020), but can also be amplified directly through either rolling circle amplification (RCA) or *in vivo* phagemid amplification (Praetorius et al., 2017; Qi et al., 2018; Jia et al., 2021). The amplified ssDNA product is then digested by restriction enzymes to separate out the ssDNA region of interest (Jia et al., 2021).

Recent advances in DNAzyme research have enabled a more elegant isolation of the desired ssDNA segment. DNAzymes have progressed such that they can now cleave targets made entirely of deoxyribonucleotides (Gu et al., 2013; Wang et al., 2014), whereas previous work used a target strand containing a single ribonucleotide where cleavage occurred (Brown et al., 2003; Tian et al., 2005; He et al., 2016; Saran and Liu, 2016). Both RCA and phagemid amplification can be used to generate a continuous strand of repeated ssOrigami-forming sequences which self-cleave into the individual origami (Jia et al., 2021). The integration of these highly specific, self-cleaving DNAzymes removes the need for separate restriction enzymes and simplifies the ssOrigami production process.

While there are a variety of techniques to generate kilobase length ssDNAs, the cost of DNA origami production at a milligram scale has the same order of magnitude of ∼$100 USD/mg for both ssOrigami and scaffold-staple systems (Jia et al., 2021). While producing milligrams of origami is a reasonable scale in a research context, industrial applications will require far larger quantities. At Gram scale production, ssOrigami (with integrated DNAzymes) can be produced cheaper, with anticipated fermenter-assisted phagemid production costs approaching $200 USD/g (Praetorius et al., 2017; Jia et al., 2021). MsOrigami production at a Gram scale continues to rely on chemical synthesis for staple production, so does not scale as efficiently. Thus, while initial research costs in a lab setting is roughly equivalent for both ssOrigami and msOrigami, mass-production of ssOrigami is more economical.

## 5 Outlook and perspective

SsOrigami is an underutilized and underexplored subfield in the world of DNA nanostructures. This seemingly simple design choice tempers several of drawbacks of more traditional msOrigami. Its ability to fold even at low concentration, its comparative stability in the presence of exonucleases, its production using cellular machinery, and its industrial cost-effectiveness all make ssOrigami an alternative worth pursuing. Preliminary applications of ssDNA origami in biological conditions taking advantage of these benefits have been performed, such as the sorting of liposomes (Jia et al., 2021). While existing applications are limited, *in vivo* biosensing and bioimaging are promising directions for ssOrigami. Additionally, with the incorporation of conditionally stable DNA interactions within the sequence, responsive ssOrigami can be designed. Benefits aside, the design limitations imposed by folding order and knotting are non-trivial barriers to entry—but these can be mitigated by clever usage of secondary structures, thermodynamic control, and topology. Additionally, benefits from DNA-based ssOrigami are shared with RNA-based ssOrigami, which has also seen recent development (Geary et al., 2014; 2021; Han et al., 2017). RNA ssOrigami has the added benefit of being genetically encoded, transcribed, and self-assembled in the same cell (Geary et al., 2014)*.* For *in vivo* applications the origami can thus be synthesized within its target cell, eliminating the need for external nanostructure production and subsequent delivery into the cell—steps which traditional msOrigami would require. This has already been applied in yeast where an RNA ssOrigami nanostructure transcribed and folded *in vivo* was used to modulate gene expression in that same cell (Pothoulakis et al., 2022). Furthermore, the development of software specific to designing RNA ssOrigami makes entry into this subfield more accessible (Geary et al., 2021). While multi-stranded systems will continue to drive many applications in DNA origami research, single-stranded origami provides a refreshing approach for the mass production and *in vivo* applications of DNA/RNA nanostructures.
